# Advanced off-gas measurement using proton transfer reaction mass spectrometry to predict cell culture parameters

**DOI:** 10.1186/1753-6561-7-S6-P14

**Published:** 2013-12-04

**Authors:** Timo Schmidberger, Robert Huber

**Affiliations:** 1 Department of biotechnology, University of Natural Resources and Life Sciences, 1180 Vienna, Austria; 2 Sandoz GmbH BU Biopharmaceuticals, 6336 Langkampfen, Austria

## Background

Mass spectrometry is a well-known technology to detect O_2 _and CO_2 _in the off-gas of cell culture fermentations. In contrast to classical mass spectrometers, the proton transfer reaction mass spectrometer (PTR MS) enables the noninvasive analysis of a broad spectrum of volatile organic compounds (VOCs) in real time. The thereby applied soft ionization technology generates spectra of less fragmentation and facilitates their interpretation. This gave us the possibility to identify process relevant compounds in the bioreactor off-gas stream in addition to O_2 _and CO_2_. In our study the PTR-MS technology was applied for the first time to monitor volatile organic compounds (VOC) and to predict cell culture parameters in an industrial mammalian cell culture process.

## Materials and methods

**Figure 1:**
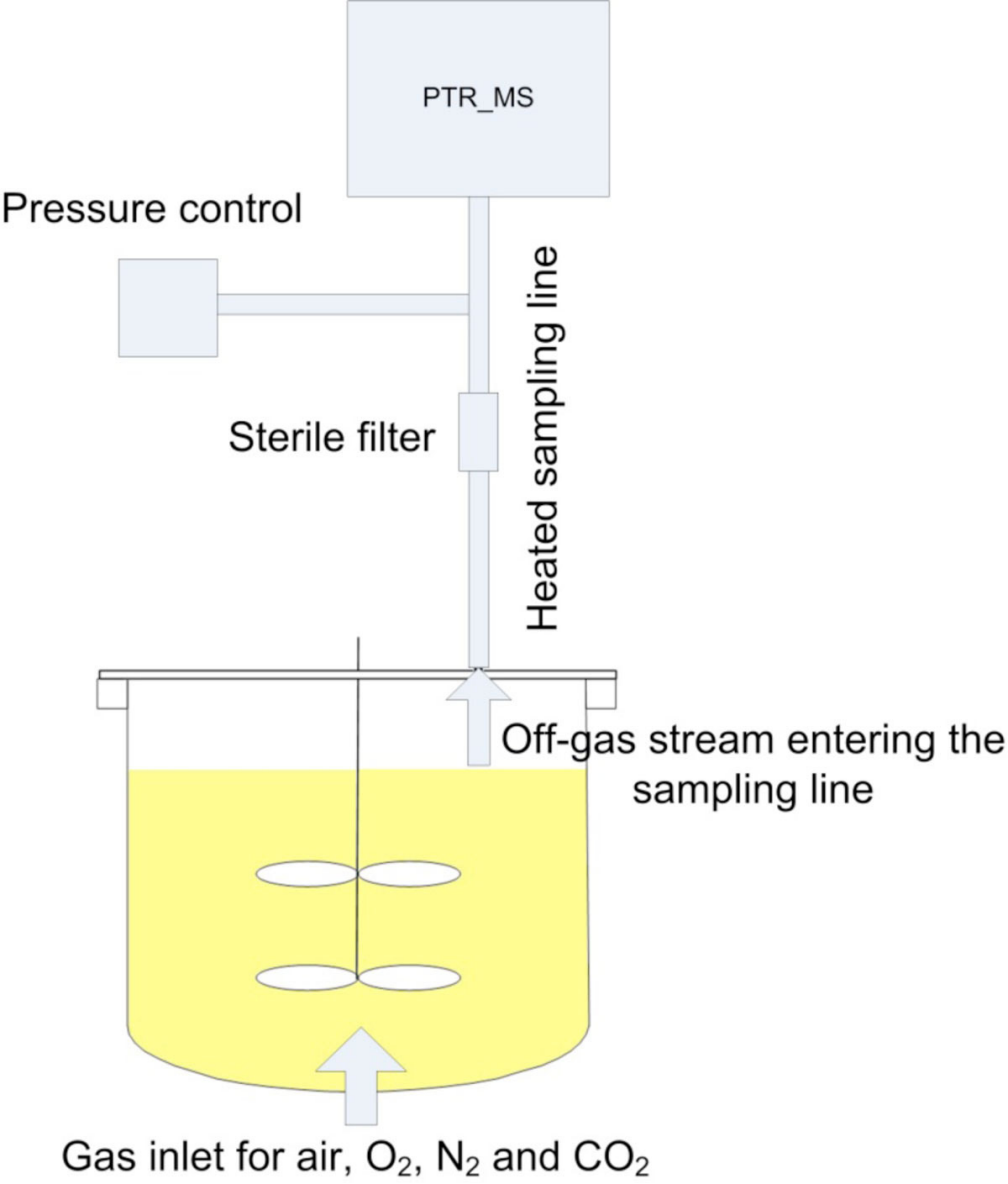
**Experimental set-up to monitor VOCs in mammalian cell culture**.

The aptitude of PTR MS for advanced bioprocess monitoring was assessed by Chinese hamster ovary (CHO) cell culture processes producing a recombinant protein conducted in a modified 7L glass bioreactor (Applikon, Shiedam, Netherlands). The PTR MS-hs (Ionicon, Innsbruck, Austria) was equipped with a QMS422 quadrupole for mass separation and with a secondary electron multiplier detector to measure masses ranging from 18 to 200m/z. The equipment set-up is illustrated in Figure 1. On a daily basis the glutamine concentration was determined with the BioProfile 100 plus (Nova Biomedical, Waltham, MA) and the viable cell density (VCD) was measured with the Vi-Cell XR cell counter (Beckham Coulter, Fullerton, CA). Samples for the product quantification were pulled daily and analyzed once at the end of a fermentation using affinity liquid chromatography. The PTR-MS data was first filtered with an adaptive online repeated median filter [1] and then correlated to the cell culture parameters with partial least square regression (PLS-R) using the software SIMCA P12+ (Umetrics, Umea, Sweden).

## Results

The applicability of the PTR-MS technique was studied using eight different fermentations conducted during process optimization to determine key cell culture parameters such as viable cell density, product titer and glutamine by partial least square regression models. Probably the most important parameter in industrial cell culture processes is the viable cell density. The R² of the PLS-R model for the VCD was 0.86 and hence, lower compared to other methods found in literature (such as 2D fluorescence [2]). Especially low values, which were observed only in the first few days of the fermentation, showed a high prediction error. At the beginning of the fermentation the VOC composition in the off-gas is characterized by VOCs from the media preparation (probably impurities of the raw materials used) and only a few VOC can be assigned to the cells. The media was prepared up to one week before the fermentations started and, depending on the storage time, the initial VOC content varied. Within the first days the media assigned VOCs were washed out and the cells started to produce VOCs. Accordingly the effect of the initial condition was weaker and prediction got better. In a second PLS-R model the product concentration was estimated based on the PTR-MS data. The model was better compared to the estimation of the VCD what is reflected in a R² of 0.94. The effect of the early process phase on the prediction quality is not very distinct since almost no product was produced in the first days. The good model for the titer is a hint that producing the product is correlated with metabolic pathways involving VOCs. However distinct metabolic pathways could not be revealed within this study, since only a few VOC could be assigned to specific compounds yet. The third parameter assessed in this study was the glutamine concentration. The PLS-R model for glutamine concentration showed the lowest R² and Q² of this evaluation. Glutamine was added on demand and probably feeding corrupted the correlation. To overcome this problem, the glutamine related physiological parameter specific glutamine uptake (qGln) was used. The descriptive as well as the predictive power was higher when the specific consumption instead of the glutamine concentration was used (0.91 and 0.82). An explanation for this result is that the consumption of glutamine might be correlated to other metabolic pathways which can produce VOCs. In combination with an accurate online VCD measurement, the qGln can be used to estimate the overall glutamine demand of the culture in real-time. A summary of all PLS-R models is given in Table 1.

**Table 1:** Summary PLS-R models

Compound	R²	Q²
VCD	0.86	0.76
Product titer	0.94	0.88
Glutamine	0.83	0.62
Specific glutamine uptake	0.91	0.82

## Conclusions

In our study we showed that the VOC profile obtained with the PTR-MS can be used to predict important cell culture parameters, but compared to other on-line techniques such as near infrared spectroscopy the PLS-R models are currently less robust (expressed by a lower R²). Moreover the most important VOCs in the PLS-R model could be used to get deeper insights into the cellular metabolism. At the moment however, this is limited by the lack of identified VOCs and the small literature basis reporting of pathways including volatile metabolites. Finally, further experiments will be necessary to assess the most influential factors on the VOC production and to fully exploit the potential of the PTR-MS.
